# Testing the PRISMA-Equity 2012 Reporting Guideline: the Perspectives of Systematic Review Authors

**DOI:** 10.1371/journal.pone.0075122

**Published:** 2013-10-10

**Authors:** Belinda J. Burford, Vivian Welch, Elizabeth Waters, Peter Tugwell, David Moher, Jennifer O’Neill, Tracey Koehlmoos, Mark Petticrew

**Affiliations:** 1 Cochrane Public Health Group and Jack Brockhoff Child Health and Wellbeing Program, Melbourne School of Population and Global Health, the University of Melbourne, Melbourne, Victoria, Australia; 2 Ottawa Hospital Research Institute, University of Ottawa, Ottawa, Canada; 3 Elizabeth Bruyere Research Institute, University of Ottawa, Ottawa, Canada; 4 Clinical Epidemiology Program, Ottawa Hospital Research Institute, Ottawa, Canada, Department of Epidemiology & Community Medicine, University of Ottawa, Ottawa, Canada; 5 Centre for Equity & Health Systems, International Centre for Diarrhoeal Disease Research (ICDDRB), Dhaka, Bangladesh, Dhaka; 6 Department of Social and Environmental Health Research, London School of Hygiene and Tropical Medicine, London, United Kingdom; Universidad Peruana de Ciencias Aplicadas (UPC), Peru

## Abstract

Reporting guidelines can be used to encourage standardised and comprehensive reporting of health research. In light of the global commitment to health equity, we have previously developed and published a reporting guideline for equity-focused systematic reviews (PRISMA-E 2012). The objectives of this study were to explore the utility of the equity extension items included in PRISMA-E 2012 from a systematic review author perspective, including facilitators and barriers to its use. This will assist in designing dissemination and knowledge translation strategies. We conducted a survey of systematic review authors to expose them to the new items in PRISMA-E 2012, establish the extent to which they had historically addressed those items in their own reviews, and gather feedback on the usefulness of the new items. Data were analysed using Microsoft Excel 2008 and Stata (version 11.2 for Mac). Of 151 respondents completing the survey, 18.5% (95% CI: 12.7% to 25.7%) had not heard of the PRISMA statement before, although 83.4% (95% CI: 77.5% to 89.3%) indicated that they plan to use PRISMA-E 2012 in the future, depending on the focus of their review. Most (68.9%; 95% CI: 60.8% to 76.2%) thought that using PRISMA-E 2012 would lead them to conduct their reviews differently. Important facilitators to using PRISMA-E 2012 identified by respondents were journal endorsement and incorporation of the elements of the guideline into systematic review software. Barriers identified were lack of time, word limits and the availability of equity data in primary research. This study has been the first to ‘road-test’ the new PRISMA-E 2012 reporting guideline and the findings are encouraging. They confirm the acceptability and potential utility of the guideline to assist review authors in reporting on equity in their reviews. The uptake and impact of PRISMA-E 2012 over time on design, conduct and reporting of primary research and systematic reviews should continue to be examined.

## Introduction

There is a recognized global commitment to health equity, defined as the absence of avoidable and unfair inequalities in health[1], and social determinants of health[2,3]. Such commitment to action requires careful evaluation of policies, strategies and programmes (hereafter referred to as ‘interventions’), so that their effects on health equity may be understood. 

Systematic reviews, in providing a comprehensive view of an evidence base, can address health equity questions, by 1) assessing effects of interventions targeted at disadvantaged populations, such as slum upgrading strategies[4], or interventions for promoting reintegration of street-connected young people[5], or 2) assessing effects of interventions aimed at reducing social gradients, such as examining the effect of tobacco control interventions on social inequalities in smoking[6], or 3) by assessing effects of interventions not aimed at reducing inequity but where it is important to understand the effects of the intervention on equity[7], such as obesity prevention in children[8]. However, despite these examples and the potential benefit of systematic reviews that include an equity analysis, at present few systematic reviews include health equity questions and those that do, often lack sufficient detail to allow readers to assess the credibility of subgroup analyses and applicability judgements[9]. 

Reporting guidelines are one mechanism that can be used to encourage more standardised and comprehensive reporting. This approach has been widely used to enhance reporting of individual trials[10,11] as well as systematic reviews and meta-analyses[12]. Building on previous work to develop PRISMA (Preferred Reporting Items for Systematic Reviews and Meta-Analyses), we have also developed a reporting guideline for equity-focused systematic reviews (PRISMA-E 2012) in order to: 1) provide structured guidance on transparently reporting these methods and results, and 2) legitimize and emphasize the importance of reporting health equity results. The PRISMA reporting guideline consists of 27 items and the Equity extension (PRISMA-E 2012) has 20 additional items (referred to below as “extension items”) focused on the reporting of considerations relevant to equity throughout the review. The guideline and its development is described elsewhere[7]. 

The uptake of reporting guidelines can be influenced by a number of factors. While factors such as endorsement by journals and funders have an important role to play, the usability and perceived need by review authors themselves will be a determining factor, particularly in terms of guidelines being used as intended rather than primarily to satisfy reporting requirements. Therefore, we designed and implemented the current study to assess perceived utility of PRISMA-E 2012 from the perspective of systematic review authors, and identify possible barriers and facilitators to its use. This will assist in designing dissemination and knowledge translation strategies. 

## Objectives

• To explore the utility of the equity extension items included in PRISMA-E 2012 from a systematic review author perspective
• To gain an understanding of potential facilitators and barriers to using PRISMA-E 2012

## Methods

We conducted a survey of systematic review authors to ‘road-test’ PRISMA-E 2012, prior to its publication[7]. The survey consisted of 14 questions divided into 5 sections (Figure S1). The first two sections were to ascertain general information about the level of systematic review and equity experience of participants, as well as information about a recent review authored by each participant (either complete or in progress). The next section focused on asking authors to apply the extension items from the reporting guideline with their recent review in mind. Each extension item was listed and authors were asked to indicate beside each item whether they intended to address (or had already addressed) that item in their systematic review. Authors were then asked to consider the extension items generally and whether having each item within a reporting guideline might change the way that they report on equity in their reviews in future. Each extension item was listed and authors were asked to choose between the following options: “I would always address this item so a checklist would make no difference”, “I may sometimes address this item, but a checklist would help to remind me”, “A checklist would make it much more likely for me to address this item”, “I do not think this item is relevant, so a checklist would make no difference”. Last, authors were asked questions that sought opinions on the overall utility of PRISMA-E 2012 including perceived facilitators and barriers to its use.

The survey was programmed in SurveyMonkey (www.surveymonkey.com) and was open for completion August-October 2012. Targeted dissemination of the survey was conducted via listserves, blogs, and websites both internal and external to systematic review organizations such as The Cochrane Collaboration and The Campbell Collaboration, with the aim of reaching a broad range of systematic review authors.

All data were exported from SurveyMonkey into Microsoft Excel for initial tabulation and calculation of proportions. Statistical tests were conducted in Stata (version 11.2 for Mac). For the section related to applying each item in PRISMA-E 2012 to an existing review, respondents were asked to consider each equity extension item and state whether they addressed that item, or intend to address it, in their review, by indicating ‘yes’, ‘no’, or ‘unsure’. Mean (±SD) proportions of ‘yes’, ‘no’, and ‘unsure’ responses across all equity extension items are reported. The proportion of ‘yes’ and ‘no’ responses for each item were compared using a one-sample binomial test to determine statistically whether the proportion of ‘yes’ and ‘no’ responses for that item were equal, after first excluding ‘unsure’ responses. 95% binomial confidence intervals for proportions of nominal response variables were calculated using the Clopper-Pearson method.

## Results

The survey was completed by 151 respondents whose experience in conducting systematic reviews was categorized as: <1 year (9.3%); 1-2 years (22.5%); 3-5 years (21.9%); 6-10 years (23.2%); >10 years (23.2%).

Reviews they were working on were at various stages from “protocol development” (12.6%), “in the process of conducting their review” (50.4%), to “already published” (37.1%). The majority were reviews of intervention effectiveness (93.4%), but of these, approximately half did not have a major focus on health equity (47.7%). There were three ways in which health equity was being examined, in assessing the effectiveness of interventions that:

1. targeted disadvantaged populations (18.5%)
2. aimed to reduce social gradients across populations (eg. interventions to reduce the social gradient in smoking) (4.0%)
3. were not aimed at reducing inequity but where it is important to understand the effects of the intervention on equity (23.2%)

Whilst the majority of respondents (67.5%; 95% CI: 59.5% to 74.9%) had either used or were planning to use the existing PRISMA statement to guide reporting of their review, some had not heard of the PRISMA statement (18.5%; 95% CI: 12.7% to 25.7%).

### Applying PRISMA-E 2012 to existing reviews

To road-test the PRISMA-E 2012 reporting guideline on their existing reviews, respondents were asked to consider each equity extension item included in PRISMA-E 2012 and state whether they addressed that item, or intend to address it (if not at that stage yet), in their review, by indicating ‘yes’, ‘no’, or ‘unsure’. For every item except for one, the most common response was ‘no’ (the mean(±SD) proportion of ‘no’ responses across all items was 59.5±7.4%, ranging from 41.7% to 68.9%). Mean proportion of ‘yes’ and ‘unsure’ responses were 29.7±6.8% (range: 21.2-45.0%) and 10.8±2.3% (6.6-15.9%) respectively. Comparing just the ‘yes’ and ‘no’ responses for each item, the proportion of ‘yes’ responses was lower for 17 out of 20 items (p-values ranged from p=0.006746 to p<0.0001). For the remaining three items, there was no significant difference between the proportion of ‘yes’ and ‘no’ responses (at a significance level of p=0.05). These items were:

• Structured Summary: Describe extent and limits of applicability to disadvantaged populations of interest
• Conclusions: Present extent and limits of applicability to disadvantaged populations of interest and describe the evidence and logic underlying those judgements
• Conclusions: Provide implications for research, practice or policy related to equity where relevant (e.g. types of research needed to address unanswered questions)

Space was also provided for free-text responses and the comments (n=19) could be categorised into two main themes to explain why respondents did not address the equity extension items in their reviews:

• equity was not a focus of the review so the items did not seem relevant
• respondents had not considered these items but might consider them for the next update of their review.

Since a high proportion of respondents were not undertaking reviews with an equity focus and the items would not necessarily have been relevant, we analysed the results separately for those who indicated that their review had an equity focus, based on the 3 categories above (n=69). The most common response was ‘yes’ for 10 items and ‘no’ for the remaining 10 items, however the proportions were more closely matched than in the full sample of respondents. There was no significant difference between the proportion of ‘yes’ and ‘no’ responses for 17 out of 20 items (at a significance level of p=0.05). For the remaining three items, the proportion of ‘yes’ responses was significantly higher than the proportion of ‘no’ responses (p-values between p=0.00022 and p<0.0001). These items were the same three items identified as having no significant difference between the proportion of ‘yes’ and ‘no’ responses in the full set of respondents. The mean proportion of ‘yes’ and ‘no’ responses across all items were 47.3+10.1% (range: 34.8 to 68.1%) and 41.8+10.0% (range: 20.3 to 55.1%) respectively. 

### Potential utility of PRISMA-E 2012 in future reviews

**Figure 1:**
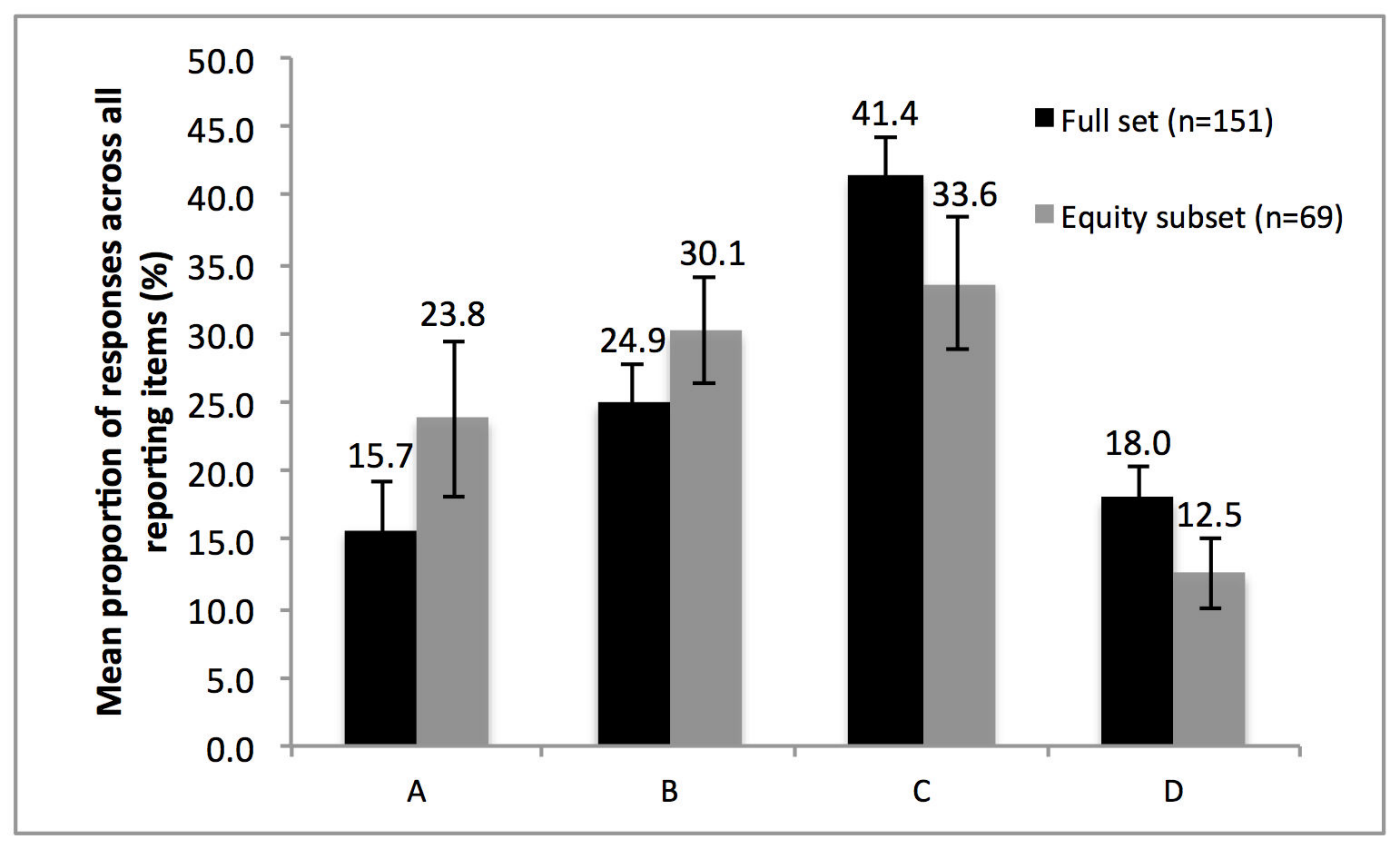
Proportion of responses (%) falling into each response category for all items in PRISMA-Equity 2012. Mean (±SD) proportion of responses (%) falling into each response category was calculated across all items in the PRISMA-Equity 2012 reporting guidelines. KEY. A. I would always address this item so a checklist would make no difference. B. I may sometimes address this item, but a checklist would help to remind me. C. A checklist would make it much more likely for me to address this item. D. I do not think this item is relevant, so a checklist would make no difference.

Respondents were asked for their opinions about the likelihood of PRISMA-E 2012 to change the way that they conduct and report reviews (in other words: how helpful did they think it would be to be reminded of the equity extension items in PRISMA-E 2012 in a reporting guideline?). To determine this, respondents were again asked to consider each equity extension item in PRISMA-E 2012 in relation to conducting reviews in future and complete one of four response options. The mean (±SD) proportions of responses in each category across all items are shown in Figure 1. The same data for the subset of respondents (n=69) who were undertaking reviews with an equity focus are also shown in Figure 1.

Overall, 83.4% (95% CI: 77.5% to 89.3%) of respondents said that they would use PRISMA-E 2012. The majority of respondents (64.9%; 95% CI: 56.7% to 72.5%) indicated that they would consider using the PRISMA-E 2012 reporting guideline depending on the focus of their review, while 18.5% (95% CI: 12.7% to 25.7%) of respondents would use the guideline for every review. The remaining respondents were either already aware of the reporting items to consider (2.0%; 95% CI: 0.4% to 5.7%), did not think the guideline was relevant (7.9%; 95% CI: 4.2% to 13.5%), or were unsure as to whether they would use it (6.6%; 95% CI: 3.2% to 11.8%). The majority responded positively about the likelihood of PRISMA-E 2012 to lead them to conduct their reviews differently (68.9%; 95% CI: 60.8% to 76.2%). Most thought that using PRISMA-E 2012 in developing their review was likely to improve the usability of their review for decisions about equity (Yes 58.3% (95% CI: 50.0% to 66.2%), No 7.9% (95% CI: 4.2% to 13.5%), Unsure 33.8% (95% CI: 26.2% to 41.9%)). Respondents could not list any additional items that they thought were missing from PRISMA-E 2012.

### Facilitators and advantages

Respondents were asked to provide comments on any perceived facilitators and advantages for them in using PRISMA-E 2012 (43 respondents answered this question). The majority of comments received mentioned advantages to use, rather than facilitators of use, and the advantages could be categorized into two main themes: 1) a reporting guideline is a helpful reminder and training tool to ensure authors are aware of the items of consider; and 2) a guideline helps to improve consistency in reporting. The facilitators mentioned were journal endorsement of PRISMA-E 2012, and incorporating relevant items into systematic review software to remind authors and enhance the ease with which information can be included within standard review reporting structures. 

### Barriers and disadvantages

Respondents were asked to provide comments on potential barriers in using PRISMA-E 2012 to guide reporting of equity in reviews. Most comments could be categorized into the following themes (n=45): 1) the length of time it would take to consider the items, 2) the number of items in the guideline, 3) likely increased length and complexity of reviews, and 4) lack of data in primary studies to enable inclusion of many of the items. 

## Discussion

This study has been the first to road-test the new PRISMA-E 2012 reporting guideline and the results provide additional insight into the way in which authors are likely to use it. Most respondents had not addressed the guideline items in developing a recent review, which points to the need for such reporting guidelines. While this was improved by analyzing the subset of respondents who considered their review to have an equity focus, the improvement was marginal. In this group, the majority of respondents had addressed three out of the 20 items in the guideline, (with no significant difference between the proportion of ‘yes’ and ‘no’ responses for the remaining 17 items) which further highlights the gap in reporting that the guideline is designed to address. 

Most respondents indicated that the reporting guideline would increase the likelihood that they would address each item in the guideline. The majority of responses fell within two of the four response categories offered (“I may sometimes address this item, but a checklist would help to remind me” or “A checklist would make it much more likely for me to address this item”). This was the case for both the full set of respondents as well as those who considered their review to have an equity focus (Figure 1), suggesting the benefit of PRISMA-E 2012 as a useful reminder. Of course it is equally important to understand whether being reminded of these items makes a real difference to reporting. In this study, 68.8% (95% CI: 60.8% to 76.2%) of respondents thought that PRISMA-E 2012 would lead them to conduct their reviews differently, suggesting we might expect to see improvements in reporting of equity in systematic reviews over time. This should be followed up by audits of equity-related reviews for the PRISMA-E 2012 items in future.

The extensive consultation that underpinned the development of PRISMA-E 2012[7] appears to have resulted in a guideline that is comprehensive and acceptable, with no additional items suggested by respondents and 83.4% (95% CI: 77.5% to 89.3%) indicating that they plan to use PRISMA-E 2012 in future. However, as intended by the developers of PRISMA-E 2012, most felt that it would only be applicable to certain reviews and should not be a uniform requirement for all reviews. 

Respondents thought that journal endorsement and incorporating the guideline elements into systematic review software would enhance the inclusion of PRISMA-E 2012 in reviews. This aligns with the views of guideline developers who, in addition to the aforementioned factors, have identified support from funding agencies and professional organisations as critical determinants of successful implementation of reporting guidelines[13]. Efforts are underway to incorporate electronic versions of reporting guideline checklists into the report generation and journal submission process. Possible barriers identified by respondents, such as time, word limits and the expectation that equity data will not be available in primary research are expected to be overcome as experience with the guideline increases and the demand for health equity information continues to influence the design and reporting of primary research studies. It should also be acknowledged that, while reviews are dependent on what has been included within primary studies, it has been found that there is often more data on equity in primary studies than is reported in systematic reviews, so there is a loss of information that occurs when moving from primary studies to systematic reviews[9]. This loss of information is one of the problems that PRISMA-E 2012 is seeking to address. 

One of the main limitations of this study is that responses are derived from a sample that actively responded to the survey request, so may not be representative of systematic review authors in general. Rather, respondents may represent a subset of authors who are particularly interested in equity and mechanisms to improve the quality and reporting of systematic reviews, and the findings should be interpreted with this in mind. Consistent with the objectives of this study, this survey aimed to establish the nature and range of responses among potential users of PRISMA-E 2012 and, while we cannot claim this sample represents the entire population of users, it is likely that those who responded to the survey will be potential users of PRISMA-E 2012. However, given that nearly 20% of respondents had not heard of PRISMA before, it is also likely that this group provides some responses that are based on seeing a PRISMA-based reporting guideline for the first time. 

Road-testing new guidelines are an important part of their development, and essential to understanding user experiences, including both usefulness and potential burden, so that reporting guidelines can be improved over time. An explanation and elaboration paper is being developed to accompany the PRISMA-E 2012 Statement paper to provide authors and reviewers with detailed information about each equity extension item and examples of good reporting from published reviews. The results of this road-testing will inform the development of the explanation and elaboration paper. We are encouraged by these early findings, and are committed to monitoring the implementation and uptake of PRISMA-E 2012 over time in order to examine the impact of the instrument on usefulness of reviews for end users, and the impact on the inclusion of equity related concepts and constructs into the design, conduct and reporting of primary research. We plan to assess uptake of this guideline by tracking the number of journals, Campbell Review Groups, and Cochrane Review Groups endorsing the reporting guideline and the number of citations to the published guideline. We also plan measure the “footprint”[14] of the reporting guideline by tracking the number of requests for support we receive (e.g. emails, phone calls) as well as indicators of PRISMA-E 2012 sharing through social networks, such as LinkedIn, Twitter, and Facebook, and the number of downloads of the Word file of the checklist on the Campbell and Cochrane Equity Method’s Groups website.

## Supporting Information

Figure S1 **Full survey distributed via SurveyMonkey.**
(PDF)
