# Supplementary material for: Testing the PRISMA-Equity 2012 Reporting Guideline: the Perspectives of Systematic Review Authors
Source: PLoS One. 2013 Oct 10;8(10):e75122. doi: 10.1371/journal.pone.0075122 (PMC3794945; doi:10.1371/journal.pone.0075122)
Supplement: Figure S1 — Full survey distributed via SurveyMonkey. (PDF) [file pone.0075122.s001.pdf]

PRISMA-E 2012 Checklist: capturing the experience of review authors

General Information

***1. Please estimate how many years experience you have in conducting systematic reviews**

2. Please estimate the length of time you have been working in the field of health equity. If you do not work inhealth equity, please select "Not applicable"

Comments

3. Please describe your training/experience related to health equity

PRISMA-E 2012 Checklist: capturing the experience of review authors

Tell us about one of your recent reviews

Consider a systematic review you are currently working on or have recently published and answer the following questions with that review in mind.

*4. At what stage is your systematic review?

*5. Which of the categories below does your review best fit into?

- My review is designed to assess effects of interventions targeted at disadvantaged populations
- My review is designed to assess effects of interventions aimed at reducing social gradients across populations (eg. interventions to reduce the social gradient in smoking)
- The intervention/s in my review are not aimed at reducing inequity, however it is important to understand the effects of the intervention/s on equity, either positively or negatively
- My review does not have a major focus on health equity (as defined by the above categories)
- My review is not a review of interventions (please describe the focus of your review, particularly in relation to equity)

*6. Did you/Do you plan to use the PRISMA statement to guide your reporting of your review?

- Yes
- No
- I have not heard of the PRISMA statement

PRISMA-E 2012 Checklist: capturing the experience of review authors

Testing out PRISMA-E 2012 on your review

Please answer the question below with your recent review in mind.

***7. Below are the extension items in the PRISMA-E 2012 checklist.**

Please indicate beside each item whether you intend to address (or have already addressed) that item in your review.

	Yes	No	Unsure
Title: Identify equity as a focus of the review, if relevant, using the term equity	○	○	○
Structured Summary: State research question(s) related to health equity	○	○	○
Structured Summary: Present results of health equity analyses (e.g. subgroup analyses or meta-regression)	○	○	○
Structured Summary: Describe extent and limits of applicability to disadvantaged populations of interest	○	○	○
Rationale: Describe assumptions about mechanism(s) by which the intervention is assumed to have an impact on health equity	○	○	○
Rationale: Provide the logic model/analytical framework, if done, to show the pathways through which the intervention is assumed to affect health equity and how it was developed	○	○	○
Objectives: Describe how disadvantage was defined if used as criteria in the review (e.g. for selecting studies, conducting analyses or judging applicability)	○	○	○
Objectives: State the research questions being addressed with reference to health equity	○	○	○
Methods: Describe the rationale for including particular study designs related to equity research questions	○	○	○
Methods: Describe the rationale for including the outcomes - e.g. how are these relevant to reducing inequity	○	○	○
Methods: Describe information sources (e.g. health, non-health, and grey sources) that were searched that are of specific relevance to address the equity questions of the review	○	○	○
Methods: Describe the broad search strategy and terms used to address equity questions of the review	○	○	○
Methods: List and define data items related to equity where such data were sought (e.g. using PROGRESS-Plus or other criteria, context)	○	○	○
Methods: Describe methods of synthesizing findings on health inequities (e.g. presenting both relative and absolute differences between groups)	○	○	○
Methods: Describe methods of additional synthesis approaches related to equity questions, if done, indicating which were pre-specified	○	○	○
Results: Present the population characteristics that relate to the equity questions across the relevant PROGRESS-Plus or other factors of interest	○	○	○
Results: Present the results of synthesizing findings on inequities	○	○	○
Results: Give the results of additional synthesis approaches related to equity objectives, if done	○	○	○
Conclusions: Present extent and limits of applicability to disadvantaged populations of interest and describe the evidence and logic underlying those judgements	○	○	○
Conclusions: Provide implications for research, practice or policy related to equity where relevant (e.g. types of research needed to address unanswered questions)	○	○	○

Additional Comments?

PRISMA-E 2012 Checklist: capturing the experience of review authors

Your opinion on the likelihood of the checklist items to change the way you...

This will help us understand what difference each item in PRISMA-E 2012 might make in the way review authors conduct reviews in general. In other words, how helpful is it to be reminded of each item in a reporting checklist?

Consider the question below in terms of your approach to conducting reviews in general, rather than any specific review.

*8. Please consider for each item whether you would usually always address that item (regardless of its inclusion in a reporting checklist), or whether you think having it in a reporting checklist would be a helpful reminder.

	I would always address this item, so a checklist would make no difference	I may sometimes address this item, but a checklist would help to remind me	A checklist would make it much more likely for me to address this item	I do not think this item is relevant, so a checklist would make no difference
Title: Identify equity as a focus of the review, if relevant, using the term equity	○	○	○	○
Structured Summary: State research question(s) related to health equity	○	○	○	○
Structured Summary: Present results of health equity analyses (e.g. subgroup analyses or meta-regression)	○	○	○	○
Structured Summary: Describe extent and limits of applicability to disadvantaged populations of interest	○	○	○	○
Rationale: Describe assumptions about mechanism(s) by which the intervention is assumed to have an impact on health equity	○	○	○	○
Rationale: Provide the logic model/analytical framework, if done, to show the pathways through which the intervention is assumed to affect health equity and how it was developed	○	○	○	○
Objectives: Describe how disadvantage was defined if used as criteria in the review (e.g. for selecting studies, conducting analyses or judging applicability)	○	○	○	○
Objectives: State the research questions being addressed with reference to health equity	○	○	○	○
Methods: Describe the rationale for including particular study designs related to equity research questions	○	○	○	○
Methods: Describe the rationale for including the outcomes - e.g. how are these relevant to reducing inequity	○	○	○	○
Methods: Describe information sources (e.g. health, non-health, and grey sources) that were searched that are of specific relevance to address the equity questions of the review	○	○	○	○
	○	○	○	○

PRISMA-E 2012 Checklist: capturing the experience of review authors

Methods: Describe the broad search strategy and terms used to address equity questions of the review	○	○	○	○
Methods: List and define data items related to equity where such data were sought (e.g. using PROGRESS-Plus or other criteria, context)	○	○	○	○
Methods: Describe methods of synthesizing findings on health inequities (e.g. presenting both relative and absolute differences between groups)	○	○	○	○
Methods: Describe methods of additional synthesis approaches related to equity questions, if done, indicating which were pre-specified	○	○	○	○
Results: Present the population characteristics that relate to the equity questions across the relevant PROGRESS-Plus or other factors of interest	○	○	○	○
Results: Present the results of synthesizing findings on inequities	○	○	○	○
Results: Give the results of additional synthesis approaches related to equity objectives, if done	○	○	○	○
Conclusions: Present extent and limits of applicability to disadvantaged populations of interest and describe the evidence and logic underlying those judgements	○	○	○	○
Conclusions: Provide implications for research, practice or policy related to equity where relevant (e.g. types of research needed to address unanswered questions)	○	○	○	○

Additional Comments?

PRISMA-E 2012 Checklist: capturing the experience of review authors

Your thoughts on the overall utility of the PRISMA-E 2012 checklist

Having now seen all the new checklist items in PRISMA-E 2012, please tell us what you think.

***9. Would you use PRISMA-E 2012 to assist in reporting equity in your systematic reviews?**

- ◯ Yes, I would use this checklist for every review
- ◯ Yes, I would consider using this checklist depending on the focus of my review
- ◯ No, I would not use this checklist because I am already aware of the items to include in reporting equity in my reviews
- ◯ No, I would not use this checklist because it is not relevant
- ◯ Unsure

Additional comments

***10. Overall, do you think using PRISMA-E 2012 would lead you to report (or conduct) your review differently?**

- ◯ Most definitely
- ◯ Possibly
- ◯ No, because I already include these items in reporting my reviews
- ◯ No, because I would not include many of these items as they are not relevant
- ◯ Unsure

Additional comments

***11. Do you think using PRISMA-E 2012 in developing your review would improve the useability of your review for decisions about equity?**

- ◯ Yes
- ◯ No
- ◯ Unsure

Additional comments

12. Please list any perceived facilitators and advantages for you in using PRISMA-E 2012 to guide reporting on equity in your reviews?

13. Please list any potential barriers for you in using PRISMA-E 2012 to guide reporting on equity in your reviews?

14. Are there any items missing from PRISMA-E 2012?
